# Optimum Nitrogen Application Acclimatizes Root Morpho-Physiological Traits and Yield Potential in Rice under Subtropical Conditions

**DOI:** 10.3390/life12122051

**Published:** 2022-12-07

**Authors:** Md. Salahuddin Kaysar, Uttam Kumer Sarker, Sirajam Monira, Md. Alamgir Hossain, Uzzal Somaddar, Gopal Saha, S. S. Farhana Hossain, Nadira Mokarroma, Apurbo Kumar Chaki, Md. Sultan Uddin Bhuiya, Md. Romij Uddin

**Affiliations:** 1 Department of Agronomy, Bangladesh Agricultural University, Mymensingh 2202, Bangladesh; 2 Department of Crop Botany, Bangladesh Agricultural University, Mymensingh 2202, Bangladesh; 3 Department of Agronomy, Patuakhali Science and Technology University, Dumki, Patuakhali 8602, Bangladesh; 4 Department of Agricultural Extension, Khamarbari, Dhaka 1215, Bangladesh; 5 Plant Physiology Division, Bangladesh Agricultural Research Institute, Gazipur 1701, Bangladesh; 6 On Farm Research Division, Bangladesh Agricultural Research Institute, Gazipur 1701, Bangladesh; 7 School of Agriculture and Food Sciences, The University of Queensland, Brisbane, QLD 4072, Australia

**Keywords:** root length, leaf area index, effective tillers, correlation matrix, yield

## Abstract

Nitrogen (N) is a highly essential macronutrient for plant root growth and grain yield (GY). To assess the relationship among N, root traits, and the yield of *boro* (dry season irrigated) rice, a pot experiment was performed in the Department of Agronomy, Bangladesh Agricultural University, Mymensingh, Bangladesh, during the *boro* rice season of 2020–2021. Three *boro* rice varieties, namely BRRI dhan29, Hira-2, and Binadhan-10, were planted at four N doses: 0 kg ha^−1^ (N_0_), 70 kg ha^−1^ (N_70_), 140 kg ha^−1^ (N_140_), and 210 kg ha^−1^ (N_210_). The experiment was conducted following a completely randomized design with three replicates. The varieties were evaluated for root number (RN), root length (RL), root volume (RV), root porosity (RP), leaf area index (LAI), total dry matter (TDM), and yield. The results indicated that the Binadhan-10, Hira-2, and BRRI dhan29 varieties produced better root characteristics under at the N_140_ and N_210_ levels. A substantial positive association was noticed between the grain yield and the root traits, except for root porosity. Based on the root traits and growth dynamics, Binadhan-10 performed the best at the N_140_ level and produced the highest grain yield (26.96 g pot^−1^), followed by Hira-2 (26.35 g pot^−1^) and BRRI dhan29 (25.90 g pot^−1^).

## 1. Introduction

Rice (*Oryza sativa*) is the most preferred regular meal by more than 50% of people worldwide [1]. An essential organ of plants is their root system. Nitrogen (N), a vital mineral component needed for crop development, is extensively utilized in crop cultivation [2]. Rice productivity is greatly influenced by the root system, which is the fundamental structure for directly using soil nutrients, nitrogen absorption, and transportation [3]. Additionally, roots provide mechanical support for plants and hormones that aid in various physiological and biochemical processes related to growth and development. A robust root system is necessary for the growth of vigorous plants and, consequently, higher productivity. N is the most essential nutrient, and its availability and internal concentration have an impact on how biomass is distributed between the roots and shoots [4]. Intermediate N levels have been shown to enhance root elongation and root contact areas, whereas root growth decreased under higher and lower fertilization levels [5]. The development and expansion of above-ground plant parts, GY, and nitrogen use efficiency (NUE) are assumed to be strongly correlated with the phenotypic and physiological root attributes [6]. Nitrogen serves as one of the most vital minerals in rice production. Chlorophyll, structural and functional proteins, and nucleotides are the biomolecules that require nitrogen as an essential component [7]. Tiller development, grain yields, and yield attributes in rice are positively associated with nitrogen [8]. In rice, nitrogen enhances the leaf area, rate of photosynthesis, dry matter production, and GY, assuming that lodging does not occur and that pests can be efficiently managed.

A significant quantity of N is necessary for crop growth, and plants that receive insufficient N exhibit symptoms such as reduced chlorophyll content, photosynthetic activity, biomass production, early leaf senescence, and decreased GY as well as quality [9]. Thus, the nutrition of rice plants, efficient nutrient–nitrogen management, and avoidance of probable climatic issues caused by nitrogen losses related to elevated nitrogen levels all depend on the soil’s capacity to supply N. In addition to water, N is a significant element in crop output [10]. The ongoing increase in the global rice yield over the last 50 years has been chiefly attributed to the rise in fertilizer nutrient supply, particularly nitrogen fertilizers [11,12]. Current agricultural practices heavily rely on the widespread application of synthetic fertilizers, which is bad for the environment and eventually lowers soil quality and plant productivity [13]. The usage of nitrogen fertilizer is typically ineffective, and on average, its apparent recovery efficiency is only 33% [14]. The sequential intake of N and P exerts the greatest impact on root development, shape, and allocation among the necessary mineral nutrients [15]. Changes in N abundance and its distribution in soil remarkably impact how roots develop [16].

Plants have developed various techniques to adjust the root absorption capability and make up for the soil’s decreasing nutrient supply [17]. Generally, the structure of the root systems of plants can alter or may be regulated depending upon compartments enriched with nutrients [18], which may significantly affect the nitrogen uptake from the soil [17]. It has been found that nitrogen, primarily in inorganic forms, controls crop root development and impacts how much soil a plant explores entirely. The intercellular nitrate (NO3^−^) and NH4^+^ levels appear to be important foreign indicators that are identified by the root system regionally [19]. In contrast, many substances such as hormones, carbohydrates, and nutrients themselves (or their metabolic products) serve as systemic inbuilt indicators for regional environments, from root to shoot and vice versa [20], notifying the plants’ root N requirements. Hence, such signaling pathways allow the plants to detect the presence of nitrogen locally, causing them to produce more N carriers and extend secondary roots in response to N [21]. Numerous investigations have proven that more extensive root systems, primarily the result of enormous root biomass and extended root length density, might allow rice cultivars to explore more soil and receive more nutrients from different depths, eventually leading to greater seed production and NUE [22,23,24].

According to prior studies, RN responded favorably to N concentrations, whereas RL and diameter did not respond well [25]. Rice roots treated with minimal N exhibited a greater entire contact area, more roots, and extended root lengths during the growing periods compared with rice roots treated with high N [26]. Root characteristics exhibit significant plasticity in rice’s adaptive responses to varying levels of N application [22,27,28]. There has been little research on the morphological characteristics of rice roots under various N treatments, such as RN, RL, and RV. This is mostly because root determination is technically challenging [27,29]. Therefore, the goal of the current study aimed to assess grain yields and root attributes of *boro* rice varieties along with their association under different nitrogen supply conditions.

## 2. Materials and Methods

### 2.1. Experimental Sites and Plant Materials

The research was performed in the net house of the Agronomy Department, Bangladesh Agricultural University, Mymensingh, Bangladesh (latitude: 24°42′55″, longitude: 90°25′47″) during the *boro* seasons of 2020–2021. The location is in the Old Brahmaputra Floodplain Agro-ecological Zone and contains non-calcareous dark grey floodplain soil (AEZ 9) [30]. Three *boro* rice varieties were used as the study materials. Seeds of BRRI dhan29 (V_1_, inbred), Binadhan-10 (V_2_, inbred), and Hira-2 (V_3_, hybrid) were collected from the Bangladesh Rice Research Institute (BRRI), Bangladesh Institute of Nuclear Agriculture (BINA), and regional market, respectively. The details of the three varieties are presented in Table 1. The weather data as documented by the Department of Irrigation and Water Management, Bangladesh Agricultural University, Mymensingh, Bangladesh are shown in Figure S2. Before starting the experiment, the physicochemical properties of the pot soils were analyzed and are stated in Table 2.

### 2.2. Experimental Design and Crop Management

A completely randomized design (CRD) was employed to perform the experiment. The collected soil was dried under the sun, followed by thorough crushing and mixing, and 25 kg of soil was placed in each of the 30 L plastic pots (35 cm diameter and 40 cm height). The fertilizers including triple super phosphate, muriate of potash, gypsum, and zinc sulphate were applied at the rate of 2.5 g, 3.25 g, 2.81 g, and 0.09 g pot^−1^, respectively [31], at the end of pot preparation. Urea served as the nitrogen source, and different levels of N were used such as the N_0_ (0 g pot^−1^), N_70_ (3.79 g pot^−1^), N_140_ (7.59 g pot^−1^), and N_210_ (11.39 g pot^−1^) levels. One-third of the urea for each treatment was broadcasted at the end of the pot preparation. The remaining amount of the urea, as per specification, was applied in two splits at 20- and 40-day intervals after transplanting. The formerly raised seedlings (40 days old) of the studied varieties were transplanted while maintaining three plants in the pot and keeping 4 cm of standing water. The plots received irrigation up to 15 days before harvest. Weeds were frequently observed throughout the growing season, particularly in the initial stages, and were manually pulled up. There was no significant insect infestation during the research period.

### 2.3. Measurement of Root Morphological and Physiological Traits

The root morphological attributes were collected at 20, 40, 60, and 80 DAT and at the harvest stage. From each pot, three plants were uprooted, and the estimated value of the different traits was averaged.

#### 2.3.1. Root Number

The plants were plucked by making a large incision around the base after being watered. Root samples were cleaned with running tap water to remove dirt from samples on 1 mm mesh screens [32]. The root number (RN) plant^−1^ was calculated by counting the roots.

#### 2.3.2. Root Length (cm)

The root length (RL) was estimated at different DATs and at the harvest stage from core samples [33]. The RL was estimated from the bottom of the plant to the tip of the central axis of the root by a centimeter scale (Figure S1) and the total value was calculated.

#### 2.3.3. Root Volume (cm^3^ hill^−1^)

The roots were gently dug up with mud and rinsed with flowing water. The root volume (RV) was calculated by putting the root biomass into a graduated cylinder that contained a given amount of water [34]. The rise in the water level was measured and represented as cm^3^ hill^−1^.

#### 2.3.4. Root Porosity (%)

The roots that were collected were soaked in water and sealed in airtight polyethylene bags to retain the precise root temperature. Both the water-filled and empty pycnometer vials were weighed. The temperature of the water-filled vial was measured. Excess water was gently drained from the root sample using tissue paper and transferred to the blotting paper. Using an analytical balance, the root weight was measured. The roots were placed into a vial that contained water. The roots were placed into the pycnometer vial with a sterilized needle to release any detected air bubbles. An analytical balance was utilized to measure the weight of water and entire fresh roots. The roots were then withdrawn from the vial and crushed using a glass pestle and mortar. The pycnometer was filled with the entire homogenate. The weight was measured once the homogenate and pycnometer reached ambient temperature. The root porosity (RP) was measured using the equation mentioned [35]:

(1) $$\%\;\mathrm{porosity}\;=\frac{W_{\mathrm{hr}+w}-W_{\mathrm{fr}+w}}{W_{w}+W_{\mathrm{fr}}-W_{\mathrm{fr}+w}}$$

where W_hr+w_ = weight of homogenized roots- and water-filled pycnometer vial, W_fr+w_ = weight of fresh roots- and water-filled pycnometer vial, W_w_ = weight of water-filled pycnometer vial, and W_fr_ = weight of fresh roots.

#### 2.3.5. Physiological Traits

For the calculation of the LAI, the leaf area (LA) was determined with the help of a leaf area meter (LI 3100, Licor, Inc., Lincoln, NE, USA). The LAI was calculated as the ratio of LA to the ground area. At different phases of development, the growth parameters—namely, the CGR equation developed by [36,37], the RGR established by [38], and the NAR estimated by the equation of [39]—were assessed.

#### 2.3.6. Total Dry Matter (TDM)

Three hills (plants) were pulled from each treatment at every developmental phase. The isolated leaves (blade), culms with sheaths, and panicles were dried in an oven and then weighed using a digital scale. The mean values (g hill^−1^) of the dry weights of the leaves, stems, and panicles were then determined, and the dry weights of the various plant parts were summed to calculate the TDM.

#### 2.3.7. Yield and Yield Components

When the grains were 90% matured, crop plants were harvested. The rice GY was estimated in g pot^−1^ at 14% moisture content. Data on yield attributes such as the plant height (PH), number of total tiller plant^−1^ (TTP), number of effective tiller plant^−1^ (ET), panicle length (PL), panicle number (PN), number of grain panicle^−1^ (GP), 1000-grain weight (TGW), GY, and straw yield (SY) of every plant were documented and then accordingly averaged. The harvest index (HI%) of the plant was calculated at the harvest stage using the yield of the grains and cumulative grain and straw yield plant^−1^ [40].

### 2.4. Statistical Analysis

The statistical package JMP Pro 16 (SAS Institute Inc) was used for the two-way analysis of variance (ANOVA) test, and the mean differences were compared through Tukey’s honestly significant difference (HSD) post hoc test at *p <* 0.05 and *p* < 0.01 probability levels. Sigma Plot v14 (Systat Software, Inc., San Jose, CA, USA, www.systatsoftware.com, (accessed on 11 August 2022)) and R (R for windows 4.1.2) software were used for the data visualization and correlation matrix.

## 3. Results

### 3.1. Root Morphological Traits, Total Dry Matter and Leaf Area Index

**Figure 1:**
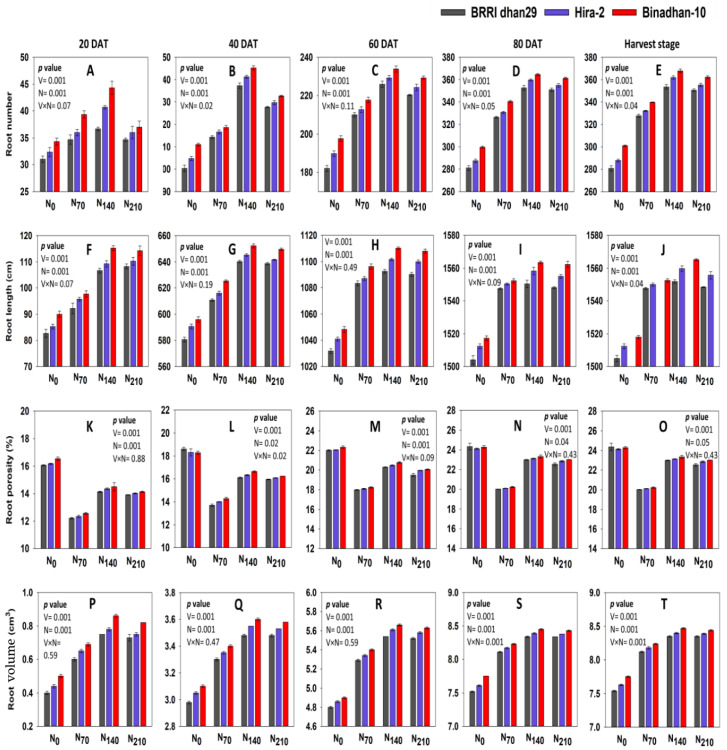
Dynamic root morphological traits of three rice varieties under four N treatments from 20 DAT to the harvest stage. N_0_: 0 kg ha^−1^, N_70_: 70 kg ha^−1^, N_140_: 140 kg ha^−1^, N_210_: 210 kg ha^−1^; (**A**–**E**) denote RN; (**F**–**J**) denote RL; (**K**–**O**) denote RP; (**P**–**T**) denote RV.

The N level strongly influenced the RN of the three rice varieties (Figure 1 and Table S1). Binadhan-10 produced the highest number of roots at 38.75, 126.92, 219.67, 341.50, and 342.75 at the 20, 40, 60, and 80 DAT stages and the harvest stage, respectively. At the N_140_ level, the highest number of roots was 40.56, 141.33, 229.78, 359.00, and 361.22, which was found at the 20, 40, 60, and 80 DAT stages and the harvest stage, respectively. The RN enhanced with the rise in N level up to N_140_; beyond that stage, the RN started to decline. Nitrogen and variety interactions have a substantial impact on the RN. The greatest RN was observed in Binadhan-10 at the N_140_ level, while the lowest value was observed in BRRI dhan29 at N_0_ at all observations. The RN at N_70_, N_140_, and N_210_ for Binadhan-10 at 80 DAT was 13.57%, 21.69%, and 20.58% higher, respectively, compared with N_0_.

Similar to RN, N substantially affected the RL of the three rice varieties (Figure 1). The highest RL was found in Binadhan-10, which was 104.33, 630.83, 1090.75, 1548.83, and 1549.83 cm at the 20, 40, 60, and 80 DAT stages and at the harvest stage, respectively. The RL increased up to the N_140_ level, then subsequently reduced with the elevated nitrogen level (N_210_). The maximum RL of 110.44, 646.00, 1101.56, 1557.39, and 1558.89 cm at the 20, 40, 60, and 80 DAT stages and the harvest stage, respectively, was found at N_140_. Binadhan-10 produced 2.30%, 3.04%, and 2.97% higher RLs at N_70_, N_140_, and N_210_ level compared with N_0_ at 80 DAT.

Roots of Binadhan-10 had the highest value of root porosity at 14.44, 16.36, 20.35, 22.71, and 22.72% at the 20, 40, 60, and 80 DAT stages and the harvest stage, respectively. The highest value of root porosity was found at N_0_ at 16.26, 18.41, 22.13, 24.24, and 24.26% at the 20, 40, 60, and 80 DAT stages and at the harvest stage, respectively. Binadhan-10 exhibited the maximum score of RP at N_0_, which was 16.54, 18.29, 22.33, 24.28, and 24.29% at the 20, 40, 60, and 80 DAT stages and the harvest stage, respectively. Conversely, BRRI dhan29 produced the lowest root porosity at N_70_, which was 12.22, 13.71, 17.99, 20.01, and 20.02% at the 20, 40, 60, and 80 DAT stages and the harvest stage, respectively.

The RV in the three rice varieties was substantially increased with the augmented nitrogen levels, but at a higher dose, it decreased irrespective of all varieties (Figure 1 and Table S2). The highest root volume was found in Binadhan-10 at 0.72, 3.42, 5.40, 8.21, and 8.23 cm^3^, consecutively noted at the 20, 40, 60, and 80 DAT stages and during the harvest stage, respectively. The highest root volume measured with N_140_ was the 0.80, 3.54, 5.61, 8.39, and 8.40 cm^3^ accordingly recorded at the 20, 40, 60, and 80 DAT stages and during the harvest stage. The root volume at N_70_, N_140_, and N_210_ for Binadhan-10 was 6.19, 9.03, and 8.77% higher, respectively, compared with N_0_ at 80 DAT.

**Figure 2:**
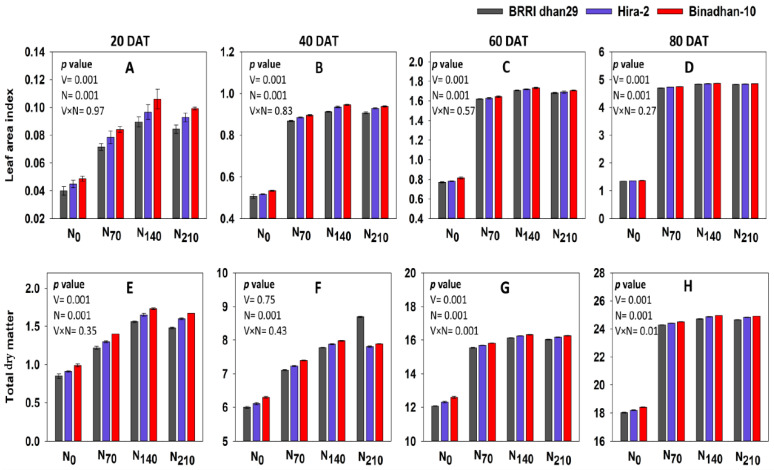
Leaf area index (LAI) and total dry matter (TDM) of three rice varieties under four N treatments from 20 DAT to 80 DAT. N_0_: 0 kg ha^−1^, N_70_: 70 kg ha^−1^, N_140_: 140 kg ha^−1^, N_210_: 210 kg ha^−1^; (**A**–**D**) denoteLAI; (**E**–**H**) denoteTDM.

The effect of nitrogen and varieties on the TDM and LAI at different DATs is presented in Figure 2 and Table S3. Binadhan-10 had the highest TDM (23.19 g plant^−1^) and LAI (3.96) at 80 DAT, whereas the lowest was found in BRRI dhan29, which had a TDM of 23.92 g plant^−1^ and LAI of 3.93 at 80 DAT. Under N_140_ treatment, the highest TDM (24.85 gplant^−1^) and LAI (4.85) at 80 DAT were obtained, whereas the lowest TDM was produced at N_0_ (18.21 g plant^−1^) and the lowest LAI (1.36) at 80 DAT. In the case of interaction, Binadhan-10 produced the highest TDM (24.97 g plant^−1^) and LAI (4.87) at 80 DAT under N_140_; BRRI dhan29 produced the lowest value of TDM (18.02 g plant^−1^) and LAI (1.35) at 80 DAT under N_0_.

### 3.2. Growth Parameters

**Figure 3:**
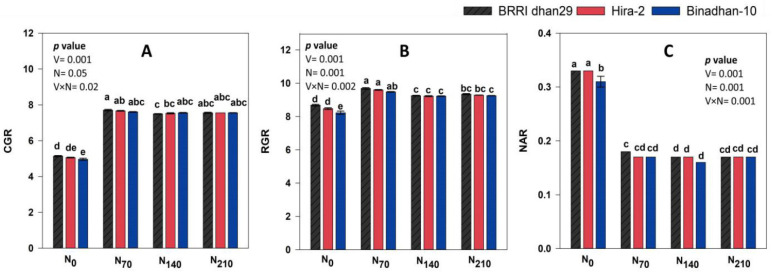
CGR, RGR, and NAR values of the three rice varieties under the four N treatments at 60–80 DAT. N_0_: 0 kg ha^−1^, N_70_: 70 kg ha^−1^, N_140_: 140 kg ha^−1^, N_210_: 210 kg ha^−1^; (**A**–**C**) denote the CGR, RGR, and NAR, respectively.

The trend of the CGR, RGR, and NAR of studied varieties under different nitrogen treatments of 60–80 DAT is presented in Figure 3 and Table S4. At the early stage, the value of the CGR was lower and peaked at 40–60 DAT, then again tended to decrease. The RGR and NAR were higher at the initial stage and tended to decrease at the later stage. At 60–80 DAT, BRRI dhan29 had the highest (6.98 g m^−2^ day^−1^) value of CGR, whereas Binadhan-10 had the lowest (6.92 g m^−2^ day^−1^) value. In relation to the N treatment, N_70_ gave the highest (7.77 g m^−2^ day^−1^) CGR, whereas N_0_ produced the lowest (5.05 g m^−2^ day^−1^) value of CGR. When interaction occurred between the variety and N, BRRI dhan29 had the highest CGR (7.71 g m^−2^ day^−1^) at N_70_, while Binadhan-10 had the lowest value (4.96 g m^−2^ day^−1^) at N_0_. At 60–80 DAT, the RGR value of BRRI dhan29 was 8.69, 9.69, 9.26, and 9.35 g m^−2^ day^−1^ at N_0_, N_70_, N_140_, and N_210_, respectively. The variety Binadhan-10 produced the RGR value of 8.23, 9.48, 9.23, and 9.26 mg g^−2^ day^−1^ at the N_0_, N_70_, N_140_, and N_210_ levels, respectively. In the interaction between the variety and nitrogen treatments, BRRI dhan29 produced the highest value of RGR (9.69 mg g^−2^ day^−1^) at N_70_ at 60–80 DAT, while Binadhan-10 produced the lowest value (8.23 mg g^−2^ day^−1^) at N_0_ level.

The net assimilation rate (NAR) of the rice varieties tended to decline after 20–40 DAT regardless of the time frames or N levels. At 60–80 DAT, BRRI dhan29 had the highest NAR (0.21 g cm^−2^ day^−1^) while Binadhan-10 had the lowest value of NAR (0.20 g cm^−2^ day^−1^). Conversely, in response to N treatment at the N_0_ level, the maximum (0.32 g cm^−2^ day^−1^) value of NAR was observed, while the N_140_ and N_210_ levels gave the lowest (0.17 g cm^−2^ day^−1^) values of RGR at 60–80 DAT. In the case of interaction, the highest (0.33 g cm^−2^ day^−1^) value of NAR was observed in BRRI dhan29 at N_0_ while the lowest (0.16 g cm^−2^ day^−1^) value was noticed in Binadhan-10 at the N_140_ level at 60–80 DAT.

### 3.3. Yield Attributing Characters and Yield

**Figure 4:**
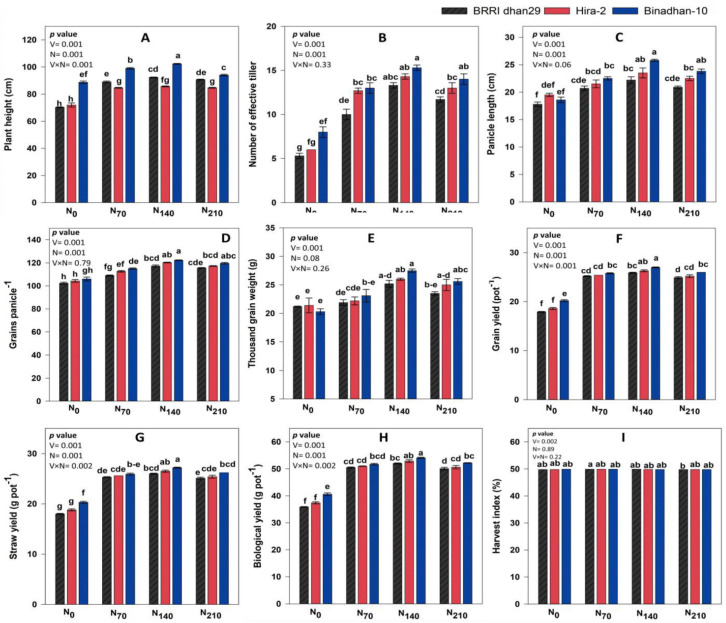
Yield and yield-contributing parameters of the three rice varieties under the four N treatments (interaction). N_0_: 0 kg ha^−1^, N_70_: 70 kg ha^−1^, N_140_: 140 kg ha^−1^, N_210_: 210 kg ha^−1^; (**A**–**E**) denote the PH, ET, Pl, GP, and TGW, respectively; (**F**–**I**) denote the GY, SY, BY, and HI, respectively.

The yield attributes and yield were influenced by variety and N, and the interaction of variety and nitrogen are presented in Table 3 and Table S5. Binadhan-10 produced the highest number of ET (12.58), PL (22.71 cm), GP (115.75), TGW (24.11 g), and GY (24.73 g pot^−1^), whereas BRRI dhan29 produced the lowest value for all of these parameters (Table 3). In the case of N application, the highest value of ET (14.33), PL (23.86 cm), GP (120.00), TGW (26.23 g), and GY (26.40 g pot^−1^) was found at the N_140_ level, whereas N_0_ produced the lowest value for the same parameters. The interaction effect of rice variety and non-yield attributes and yield was also significant (Figure 4). The highest value of ET (15.33), PL (25.82 cm), GP (122.33), TGW (27.52 g), and GY (26.96 g pot^−1^) was noticed in Binadhan-10 at N_140_, whereas the minimum values for the studied traits were found in BRRI dhan29 at the N_0_ level (Figure 4). For Biandhan-10, it was noticed that GY at N_70_, N_140_, and N_210_ was 27.45, 33.33, and 28.54% higher, respectively, than that with no N fertilizer. In the case of BRRI dhan29, the GY at N_70_, N_140_, and N_210_ was 40.69, 44.77, and 39.30% greater compared with N_0_, respectively. For Hira-2, the GY at N_70_, N_140_, and N_210_ was 36.39, 41.44, and 35.37% greater compared with the N_0_ level, respectively. The GY plant^−1^ increased with the increase in N rate irrespective of all varieties up to N_140_ level, where afterwards it reduced with elevated N level.

### 3.4. Relationship among Root Traits, Growth Parameters, Yield Attribute and Yield

**Figure 5:**
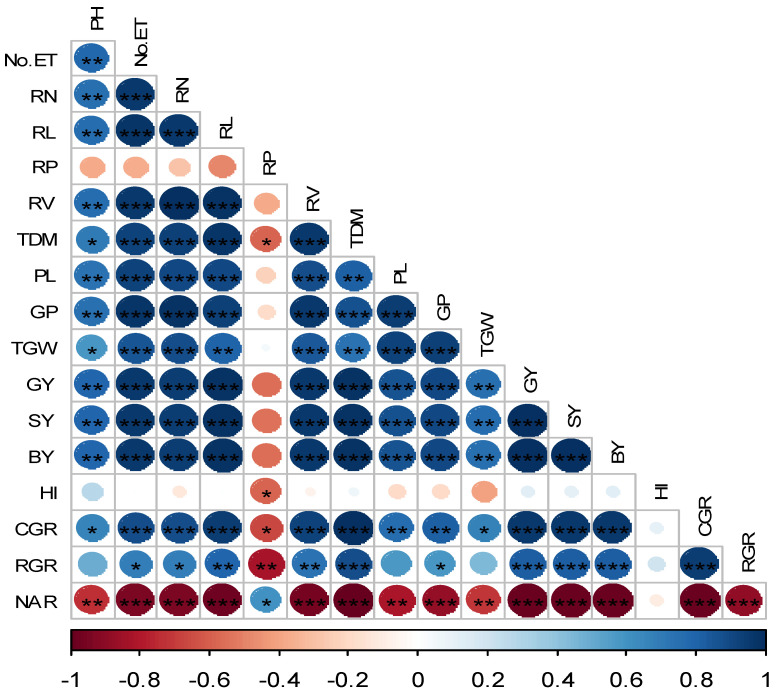
Correlation matrix of the root traits, growth parameters, yield attributes, and yield. The blue and red ellipses represented the positive and negative associations, respectively. Higher hue intensity reflects a higher co-efficient. *, ** and *** indicate the levels of significance at the 5, 1 and 0.01% level of probability. Traits details: PH, plant height; No. ET, number of effective tillers; RN, root number; RL, root length; RP, root porosity (%); RV, root volume; TDM, total dry matter; PL, panicle length; GP, grains per panicle; TGW, thousand-grain weight; GY, grain yield; SY, straw yield; BY, biological yield; HI, harvest index; CGR, crop growth rate; and NAR, net assimilation rate.

The correlation matrix of the root traits, growth parameters, yield, and yield attributes is displayed in Figure 5 to explore the association among them. The GY is significantly and positively associated with all root attributes, excluding RP. The CGR and RGR had a significant and positive connection to all root attributes and yield except for RP, while the relationship between the NAR and the root traits was significantly negative. Yield-attributing parameters such as ET, PL, GP, and TGW were also positively and substantially associated with RN, RV, and RL. Root traits also had a significant and positive correlation with TDM.

## 4. Discussion

N significantly influenced rice root development and crop yield [41]. The root is an essential rice organ that serves various physiological purposes. Dry matter and grain yield are directly correlated with root morphological characteristics [29,42]. Thus, there was a link between the rice root, nitrogen, and yield. In our experiment, the performance of the rice varieties varied under different N applications. The root characteristics, crop growth, and TDM changes may be connected to the performance differential.

When measuring the photosynthetic system, the leaf area index is utilized for determining the BY as well as GY, and a greater LAI results in a larger yield [43]. According to the present investigation, the LAI peaked at 80 DAT at the N_140_ level regardless of all varieties and afterwards started to decline due to leaf senescence. A progressive increment in the LAI might be caused by the inclusion of N, which induces the leaf number per plant and independent leaf extension. An increased N application may boost TDM levels by producing photosynthates via foliage which serves as the focal point of crop development throughout the vegetative phase and subsequent allocation of photosynthates towards genital areas [44]. A significant effect of N on the LAI was also found [45]. One of the most crucial elements for crop growth is N, a key ingredient in protein and chlorophyll, which are directly related to leaf growth [46].

Crop yields can be affected by variables including the CGR, RGR, NAR, and distribution of entire assimilates into sinks with and without economic value. The CGR trend for rice cultivars with various levels of N showed that up to the N_140_ level, it increased with the increment of plants’ age irrespective of all tested varieties; at the N_210_ level, it tended to decrease. It is logical to predict that the treatments with greater LAIs will have higher CGRs as leaves serve as the major engine of photosynthetic activities and dry matter increases per unit space, as shown by the experiment’s findings. A reduced CGR occurs during the early phases of rice cultivars because of decreased leaf growth, which is the fundamental unit of photosynthetic activities that determines the growth rate [47]. In this study, early in the crop’s growth, the maximal RGR was observed. As time went on, it progressively decreased under all N treatments and varieties. Similar RGR changes were reported under different N fertilizer levels [43]. In this study, under all N treatments, the tested varieties showed a diminishing trend with respect to the NAR value. At a greater LAI, the NAR is reduced with the increase in respiration. Reduced leaf production at the advanced stages of plant development may be related to the decreased NAR [44]. Additionally, a higher nitrogen application speeds up foliar productivity, leading to leaf shadowing due to faster canopy closure, which subsequently limits the solar energy absorption by the leaf. Hence, reduced photo-assimilates are produced, which decreases the NAR. In rice, the N fertilization with high doses was shown to decrease the value of the NAR as reported by Singh et al. [43], Esfahani et al. [48], and Yang et al. [49], which is consistent with our result.

Due to the improved yield attributes, especially for N, these are required to increase the dry matter. The TDM in rice was enhanced with the increase in N application [50,51]. The TDM was found to be elevated in our research up to the N_140_ level and afterward tended to decline. Increased N availability can enhance the TDM by producing photosynthates via leaf, which serves as the core of crop development throughout the vegetative phases and subsequent photosynthates allocation towards the breeding organelles [44,52]. Additionally, rice’s ability to produce dry matter is strongly correlated with its ability to absorb photosynthetically active radiation (PAR) [53]. Leaves with reduced N levels were the main constraint to biomass production and efficacy of irradiation, which led to decreased dry matter synthesis in rice [54]. More leaf area is enhanced by nitrogen fertilizer, which causes greater photosynthates and, further, greater dry matter formation [55]. Substantial relationships were found among the concentration of N, TDM, and GY [56].

Optimal N treatment positively influenced root development, which is consistent with earlier research showing that N accessibility had a substantial impact on root formation [57] and root enlargement [58]. The RL was also found to increase with adequate nitrogen application [5], while greater and lower N rates were found to impede root growth [59]. In this study, RN rapidly increased up to 80 DAT irrespective of all treatments and varieties. The RN significantly varied with different N treatments. According to the present findings, the RN for the varieties also increased with the augmentation of N fertilization doses and peaked at the N_140_ level. Previous research observed that the RN had a positive relationship with the N concentration [25], which is consistent with our result. The study revealed that the root development of rice varieties tended to decline when exposed to excess N (N_210_). This occurred due to ammonium toxicity from excess N fertilization [60]. Earlier studies also concluded that rice roots would grow more vigorously with optimum N management compared with excess N management [26,61].

Multiple root metrics from rice varieties subjected to various N levels were assessed [62] and disclosed that the RL increased with moderate N concentrations. Root elongation is substantially governed by the presence of N, while greater and lesser N concentrations are found to inhibit the root elongation [59]. In our study, Binadhan-10 produced the maximum RL followed by Hira-2 and BRRI dhan29 under all N treatments and observations. The RV increased by applying N at a particular level [63]. In this study, at the N_140_ level, the maximum RV was obtained irrespective of all varieties and observations, but at a higher dose of N, the RV decreased.

Data on the RP are useful for comparing assessments of rhizosphere situations, species, or variety adjustment to O_2_-confined conditions, as well as for supplying vital components to root respiration models, which use porosity to divide inbuilt diffusion between liquid and gas pathways in the root [64,65]. In our study, at the N_0_ level, cultivars produced the highest root porosity irrespective of all observations. This may be the capacity of varieties to survive the adverse situation. In this case, Binadhan-10 performed best, followed by Hira-2 and BRRI dhan29. In high land crops, aerenchyma development is stimulated by N insufficiency [66].

The recent investigation has convincingly shown that the ET and GP were directly connected to the GY. In compliance with the outcomes of our research, the hypothesis was that the ET has a significant impact on seed yields [67]. Various studies regarding the influence of N on rice yield revealed that the GY significantly increased to an extent with an increase in N content [68,69,70,71]. In our study, the GY reached a peak at the N_140_ level irrespective of all varieties; at the N_210_ level, it was decreased. Many of the earlier investigations also revealed that the higher GY values of rice are not influenced by an excessive N application rate [72,73,74]. The risk of lodging was shown to increase due to the overuse of N fertilizers, which might significantly reduce the GY [75]. However, in our study, lodging is not a major consideration that prompted the minimum output under increased N rates, as no lodging happened in this study. Increased N fertilization doses under the latest research led to decreased yields for a number of reasons. The overuse of nitrogenous fertilizers resulted in excessively luxuriant rice leaf growth [76] and thereby aged foliage is almost entirely covered by new top foliage, which may reduce the effectiveness of photosynthesis.

The correlation between the root traits and yield attribute parameters showed that the root parameters significantly influence the yield and yield attributes. When the root parameters studied in this experiment were higher, the yield contributing parameters were also higher. In this study, the GY exhibited an extremely positive and substantial association with the RL, RN, RV, PL, GP, and TGW in the studied varieties. Similarly, the RL, RN, RV, PL, and TGW were significantly and positively correlated with the GY in their studied cultivars [77]. Moreover, the results showed that the enhancement of rice yields involved superior root properties [78]. Finally, it can be concluded that in the agricultural production sector, the variety-specific fertilizer demands must be considered when setting fertilization dosages, enabling for the feasibility of maximizing rice yields by controlling the rice root growth with adequate N management.

## 5. Conclusions

Our findings indicated that N substantially affected root traits and yield. Among the three varieties, Binadhan-10 exhibited the greatest values of root traits at the 140 kg N ha^−1^ level, followed by Hira-2 and BRRI dhan29. In the case of yield and yield attributes, Binadhan-10 showed the best performance at the 140 kg N ha^−1^ level. The root traits and the yield attributes were positively correlated. From the results, it was observed that at a certain level of N dose, the root traits, yield, and yield attributes increase; however, after that particular dose, it decreased. It can be concluded that an optimum dose of nitrogen can greatly influence the root traits of rice and also has a significant influence on the yield.

## Figures and Tables

**Table 1:** List of the three rice varieties with their genetic origins and parental sources that were used in this study for root characteristics and yield.

Sl. No	Cultivar	Genetic Origin	Parental Source/Accession Number	Source
1.	BRRI dhan29	Inbred	BG90-2 × BR51-46-5	BRRI
2.	Binadhan-10	Inbred	IR42598-B-B-B-B-12 × Nona Bokra	BINA
3.	Hira-2	Hybrid	-	Local market

**Table 2:** Physicochemical properties of the soil in the pot before starting the experiment.

Soil Characteristics	Values
Soil texture	Clay loam
Soil pH	6.15
Electric conductivity (µs/cm)	641
Organic carbon (%)	1.211
Total nitrogen (N) (%)	0.110
Available form of phosphorus (P) (ppm)	28.8
Available form of potassium (K) (ppm)	83.51
Available form of sulfur (S) (ppm)	25.78

**Table 3:** Yield attributes of the three rice varieties under the four N treatments.

Variety (V)	PH (cm)	ET (no.)	PL (cm)	GP (No.)	TGW (g)	GY (g pot^−1^)	SY (g pot^−1^)	HI (%)
V_1_	85.58 b	10.08 c	20.42 b	111.08 c	22.96 b	23.47 c	23.63 c	49.83
V_2_	81.75 b	11.50 b	21.76 ab	113.67 b	23.65 ab	23.90 b	24.06 b	49.84
V_3_	96.00 a	12.58 a	22.71 a	115.75 a	24.11 a	24.73 a	24.90 a	49.84
Nitrogen (N)								
N_0_	77.00 b	6.44 c	18.66 c	104.22 d	20.98 d	18.91 c	19.03 c	49.84 a
N_70_	90.89 a	11.89 b	21.60 b	112.22 c	22.38 c	25.45 b	25.59 b	49.87 a
N_140_	93.44 a	14.33 a	23.86 a	120.00 a	26.23 a	26.40 a	26.58 a	49.84 a
N_210_	89.78 a	12.89 b	22.40 b	117.56 b	24.69 b	25.38 b	25.58 b	49.80 b
ANOVA								
V	**	**	**	**	*	**	**	NS
N	**	**	**	**	**	**	**	**
CV (%)	1.26	6.71	3.88	1.24	5.05	1.18	1.23	0.06

Within each column, the means followed by the same letters were not significantly different. **, *, and ns indicate significance at the 0.01 level, 0.05 level, and non-significance, respectively, based on the analysis of variance results. V_1_—BRRI dhan29, V_2_—Binadhan-10, and V_3_—Hira-2.

## Data Availability

Data sets analyzed during the present study are accessible from the current author upon reasonable request.

## Supplementary Materials

The following supporting information can be downloaded at: https://www.mdpi.com/article/10.3390/life12122051/s1, Figure S1: Root length measurements showing variation among varieties and N conditions; Figure S2: Weather condition during the crop growth stages of *boro* rice; Table S1: Root number and root length of three rice varieties under four N treatments from 20 DAT to harvest stage as obtained from the ANOVA analysis, Table S2: Root volume and root porosity of three rice varieties as influenced by N treatments from 20 DAT to the harvest stage as extracted from the ANOVA analysis; Table S3: Leaf area index and total dry matter of three rice varieties under four N treatments from 20 DAT to 80 DAT as obtained from the ANOVA analysis; Table S4: Crop growth rate, relative growth rate, and net assimilation rate of three rice varieties as influenced by four N treatments at 60–80 DAT acquired from the ANOVA analysis, Table S5: Yield and yield-contributing parameters of three rice varieties under four N treatments attained from the ANOVA analysis.
