# Supplementary material for: Optimum Nitrogen Application Acclimatizes Root Morpho-Physiological Traits and Yield Potential in Rice under Subtropical Conditions
Source: Life (Basel). 2022 Dec 7;12(12):2051. doi: 10.3390/life12122051 (PMC9786123; doi:10.3390/life12122051)
Supplement: Supplementary file 1 [file life-12-02051-s001.zip › Supplementary Tables.pdf]

Table S1. RN and RL of three rice varieties under four N treatments from 20 DAT to harvest stage took out from ANOVA

Variety	RN					RL (cm)				
	Days after transplanting (DAT)					Days after transplanting (DAT)				
	20	40	60	80	At harvest	20	40	60	80	At harvest
V_1	34.25 b	119.92 c	209.58 c	327.75 c	328.17 c	97.46 c	617.58 c	1074.50 c	1537.44 c	1538.08 c
V_2	36.25 ab	123.08 b	214.00 b	333.17 b	334.25 b	100.17 b	623.42 b	1082.42 b	1543.98 b	1544.44 b
V_3	38.75 a	126.92 a	219.67 a	341.50 a	342.75 a	104.33 a	630.83 a	1090.75 a	1548.83 a	1549.83 a
$S\bar{x}$	1.30	2.02	2.92	4.00	4.23	2.00	3.83	4.69	3.30	3.40
Nitrogen										
N_0	32.56 d	105.33 d	189.78 d	289.33 c	289.78 c	86.00 c	589.11 c	1040.33 c	1511.22 c	1511.72 c
N_{70}	36.67 b	116.56 c	213.44 c	332.44 b	333.11 b	95.17 b	617.33 b	1088.89 b	1549.94 b	1549.97 b
N_{140}	40.56 a	141.33 a	229.78 a	359.00 a	361.22 a	110.44 a	646.00 a	1101.56 a	1557.39 a	1558.89 a
N_{210}	35.89 b	130.00 b	224.67 b	355.78 a	356.11 a	111.00 a	643.33 a	1099.44 a	1555.11 ab	1555.89 a
$S\bar{x}$	1.64	7.84	8.89	16.06	16.28	6.11	13.29	14.34	10.84	10.96
Nitrogen : Variety										
N_0:V_1	31.00 f	100.33 j	182.00 h	281.00 i	280.67 j	82.67 f	580.67 i	1031.67 j	1504.00 h	1504.83 h
N_0:V_2	32.33 ef	104.67 i	189.67 g	287.33 h	287.67 i	85.33 f	590.67 h	1041.00 i	1512.33 g	1512.33 g
N_0:V_3	34.33 de	111.00 h	197.67 f	299.67 g	301.00 h	90.00 e	596.00 g	1048.33 h	1517.33 f	1518.00 f
N_{70}:V_1	34.67 c-e	114.33 g	210.00 e	326.33 f	327.67 g	92.17 de	610.67 f	1083.33 g	1547.42 e	1547.50 e
N_{70}:V_2	36.00 cd	116.67 fg	212.67 e	330.67 e	332.00 f	95.67 cd	616.00 e	1087.00 fg	1550.25 de	1549.92 de
N_{70}:V_3	39.33 b	118.67 f	217.67 d	340.33 d	339.67 e	97.67 c	625.33 d	1096.33 cd	1552.17 de	1552.50 cd
N_{140}:V_1	36.67 cd	137.33 c	226.00 bc	352.67 c	353.67 cd	106.67 b	640.33 c	1092.67 de	1550.33 de	1551.67 c-e
N_{140}:V_2	40.67 b	141.33 b	229.33 b	359.67 b	362.00 b	109.33 b	645.33 b	1101.67 b	1558.33 bc	1559.83 b
N_{140}:V_3	44.33 a	145.33 a	234.00 a	364.67 a	368.00 a	115.33 a	652.33 a	1110.33 a	1563.50 a	1565.17 a
N_{210}:V_1	34.67 c-e	127.67 e	220.33 d	351.00 c	350.67 d	108.33 b	638.67 c	1090.33 ef	1548.00 e	1548.33 de
N_{210}:V_2	36.00 cd	129.67 e	224.33 c	355.00 c	355.33 c	110.33 b	641.67 bc	1100.00 b	1555.00 cd	1555.67 c
N_{210}:V_3	37.00 c	132.67 d	229.33 b	361.33 ab	362.33 b	114.33 a	649.67 a	1108.00 a	1562.33 ab	1563.67 ab
$S\bar{x}$	1.05	4.20	4.83	8.57	8.71	3.31	7.14	7.76	5.86	5.92
ANOVA										
Variety (V)	**	**	**	**	**	**	**	**	**	**
N rate (N)	**	**	**	**	**	**	**	**	**	**
V x N	*	*	*	*	*	*	*	*	*	*
CV (%)	3.77	1.21	1.10	0.69	0.72	2.17	0.36	0.25	0.18	0.16

Means with the same letters within the same column do not differ significantly.

* = Significant at 5% level of probability, ** = Significant at 1% level of probability

V_1= BRRI dhan29, V_2= Hira-2, V_3= Binadhan-10

N_0= 0 Kg N ha-1, N_{70}= 70 Kg N ha^{-1}, N_{140}= 140 Kg N ha^{-1}, N_{210}= 210 Kg N ha^{-1}

Table S2. RV and RP of three rice varieties as influenced by N treatments from 20 DAT to harvest stage extracted from ANOVA

Variety	RV (cm^3/hill)					RP (%)				
	Days after transplanting (DAT)					Days after transplanting (DAT)				
	20	40	60	80	At harvest	20	40	60	80	At harvest
V_1	0.62 c	3.31 c	5.29 c	8.08 c	8.09 c	14.08 b	16.10 b	19.95 c	22.47 b	22.48 b
V_2	0.66 b	3.37 b	5.35b	8.14 b	8.15 b	14.22 b	16.19 ab	20.15 b	22.55 ab	22.56 ab
V_3	0.72 a	3.42 a	5.40 a	8.21 a	8.23 a	14.44 a	16.36 a	20.35 a	22.71 a	22.72 a
$S\bar{x}$	0.03	0.03	0.03	0.04	0.04	0.10	0.08	0.12	0.07	0.07
Nitrogen										
N_0	0.45 c	3.04 c	4.85 c	7.63 c	7.64 c	16.26 a	18.41 a	22.13 a	24.24 a	24.26 a
N_{70}	0.64 b	3.35 b	5.34 b	8.17 b	8.18 b	12.38 d	14.00 d	18.10 d	20.11 d	20.12 d
N_{140}	0.80 a	3.54 a	5.61 a	8.39 a	8.40 a	14.33 b	16.36 b	20.52 b	23.14 b	23.15 b
N_{210}	0.77 a	3.53 a	5.58 a	8.38 a	8.39 a	14.02 c	16.09 c	19.83 c	22.80 c	22.81 c
$S\bar{x}$	0.08	0.12	0.17	0.18	0.18	0.80	0.90	0.83	0.88	0.88
Nitrogen : Variety										
N_0:V_1	0.40 j	2.98 j	4.80 j	7.52 i	7.54 j	16.07 b	18.62 a	22.02 b	24.34 a	24.37 a
N_0:V_2	0.44 i	3.05 i	4.86 i	7.61 h	7.63 i	16.18 b	18.32 a	22.06 b	24.11 a	24.13 a
N_0:V_3	0.50 h	3.10 h	4.90 h	7.75 g	7.75 h	16.54 a	18.29 a	22.33 a	24.28 a	24.29 a
N_{70}:V_1	0.60 g	3.30 g	5.29 g	8.11 f	8.12 g	12.22 g	13.71 e	17.99 h	20.01 e	20.02 e
N_{70}:V_2	0.65 f	3.35 f	5.34 f	8.17 e	8.18 f	12.35 fg	14.01 de	18.09 gh	20.10 e	20.11 e
N_{70}:V_3	0.69 e	3.40 e	5.40 e	8.23 d	8.24 e	12.57 f	14.28 d	18.23 g	20.22 e	20.22 e
N_{140}:V_1	0.75 d	3.48 d	5.54 d	8.34 c	8.35 d	14.13 de	16.10 c	20.30 d	22.98 bc	22.99 bc
N_{140}:V_2	0.78 c	3.55 c	5.61 b	8.39 b	8.40 c	14.35 cd	16.33 bc	20.49 d	23.12 bc	23.13 bc
N_{140}:V_3	0.86 a	3.60 a	5.66 a	8.45 a	8.47 a	14.50 c	16.64 b	20.78 c	23.32 b	23.35 b
N_{210}:V_1	0.73 d	3.48 d	5.52d	8.34 c	8.35 d	13.91 e	15.95 c	19.48 f	22.55 d	22.55 d
N_{210}:V_2	0.75 d	3.53 c	5.58c	8.38 b	8.39 c	14.02 e	16.09 c	19.96 e	22.85 cd	22.86 cd
N_{210}:V_3	0.82 b	3.58 b	5.63 b	8.43 a	8.44 b	14.13 de	16.23 c	20.06 e	23.00 bc	23.01 bc
$S\bar{x}$	0.04	0.06	0.09	0.10	0.10	0.42	0.47	0.44	0.46	0.46
ANOVA										
Variety (V)	**	**	**	**	**	**	*	**	*	*
N rate (N)	**	**	**	**	**	**	**	**	**	**
V x N	*	*	*	**	**	*	*	*	*	*
CV (%)	2.53	0.41	0.31	0.18	0.21	1.24	1.29	0.63	0.97	1.01

Means with the same letters within the same column do not differ significantly.

* = Significant at 5% level of probability** = Significant at 1% level of probability

V_1= BRRI dhan29, V_2= Hira-2, V_3= Binadhan-10

N_0= 0 Kg N ha-1, N_{70}= 70 Kg N ha^{-1}, N_{140}= 140 Kg N ha^{-1}, N_{210}= 210 Kg N ha^{-1}

Table S3. LAI and TDM of three rice varieties under four N treatments from 20 DAT to 80 DAT obtained from ANOVA

Variety	LAI				TDM (g plant^{-1})			
	Days after transplanting (DAT)				Days after transplanting (DAT)			
	20	40	60	80	20	40	60	80
V_1	0.07 c	0.80 c	1.44 c	3.93 c	1.28 c	7.40	14.95 c	22.92 c
V_2	0.08 b	0.82 b	1.45 b	3.95 b	1.37 b	7.26	15.11 b	23.07 b
V_3	0.09 a	0.83 a	1.47 a	3.96 a	1.45 a	7.39	15.25 a	23.19 a
$S\bar{x}$	0.01	0.01	0.01	0.01	0.05	0.05	0.09	0.08
Nitrogen								
N_0	0.04 c	0.52 c	0.79 d	1.36 c	0.92 c	6.14 c	12.33 c	18.21 c
N_{70}	0.08 b	0.88 b	1.63 c	4.72 b	1.30 b	7.25 b	15.69 b	24.40 b
N_{140}	0.10 a	0.93 a	1.72 a	4.85 a	1.65 a	7.88 a	16.24 a	24.85 a
N_{210}	0.09 a	0.93 a	1.69 b	4.84 a	1.58 a	8.13 a	16.16 a	24.80 a
$S\bar{x}$	0.01	0.10	0.22	0.86	0.17	0.44	0.93	1.62
Nitrogen : Variety								
N_0:V_1	0.04 f	0.51 h	0.77 h	1.35 h	0.85 j	6.00 d	12.08 j	18.02 k
N_0:V_2	0.04 f	0.52 h	0.78 h	1.36 g	0.91 i	6.11 d	12.32 i	18.20 j
N_0:V_3	0.05 f	0.53 g	0.81 g	1.37 g	0.99 h	6.30 cd	12.60 h	18.40 i
N_{70}:V_1	0.07 e	0.87 f	1.62 f	4.70 f	1.22 g	7.11 bc	15.55 g	24.29 h
N_{70}:V_2	0.08d e	0.89 e	1.63 ef	4.73 e	1.30 f	7.23 b	15.69 f	24.41 g
N_{70}:V_3	0.08d e	0.90 de	1.64 e	4.75 d	1.40 e	7.40 b	15.83 e	24.50 f
N_{140}:V_1	0.09 b-d	0.91 c	1.71 bc	4.84 b	1.56 c	7.78 ab	16.14 c	24.72 d
N_{140}:V_2	0.10 ab	0.94 ab	1.72 ab	4.85 ab	1.65 b	7.88 ab	16.25 ab	24.86 bc
N_{140}:V_3	0.11 a	0.95 a	1.73 a	4.87 a	1.73 a	7.98 ab	16.32 a	24.97 a
N_{210}:V_1	0.08 de	0.91 c	1.68 d	4.83 c	1.48 d	8.69 a	16.03 d	24.66 e
N_{210}:V_2	0.09 b-d	0.93 b	1.69 cd	4.85 ab	1.60 c	7.81 ab	16.18 bc	24.82 c
N_{210}:V_3	0.10 ab	0.94 ab	1.71 bc	4.86 ab	1.67 b	7.89 ab	16.26 ab	24.90 b
$S\bar{x}$	0.01	0.05	0.12	0.45	0.09	0.24	0.49	0.85
ANOVA								
Variety (V)	**	**	**	**	**	NS	**	**
N rate (N)	**	**	**	**	**	**	**	**
V x N	*	*	*	*	*	*	**	**
CV (%)	8.23	0.89	0.75	0.23	1.74	0.92	0.33	0.13

Means with the same letters or without letters within the same column do not differ significantly.

* = Significant at 5% level of probability, * = Significant at 5% level of probability, NS= Non-significant

V_1= BRRI dhan29, V_2= Hira-2, V_3= Binadhan-10

N_0= 0 Kg N ha-1, N_{70}= 70 Kg N ha^{-1}, N_{140}= 140 Kg N ha^{-1}, N_{210}= 210 Kg N ha^{-1}

Table S4. CGR, RGR and NAR of three rice varieties as influenced by four N treatments at 60-80 DAT acquired from ANOVA

Variety	CGR (g m^{-2} day^{-1})			RGR (mg g^{-1} day^{-1})			NAR (g cm^{-2} day^{-1})		
	1st	2nd	3rd	1st	2nd	3rd	1st	2nd	3rd
V_1	6.03	7.06	6.98a	38.46a	15.40	9.25a	1.23a	0.42	0.21a
V_2	5.80	7.37	6.96ab	36.74b	15.89	9.15b	1.14b	0.43	0.21a
V_3	5.85	7.37	6.92b	35.79b	15.70	9.05c	1.11b	0.42	0.20b
$S\bar{x}$	0.07	0.10	0.02	0.78	0.14	0.06	0.04	0.01	0.01
Nitrogen									
N_0	5.16c	5.78b	5.05c	41.29a	15.15b	8.47c	1.57a	0.56a	0.32a
N_{70}	5.86b	7.96a	7.66a	37.28b	16.77a	9.59a	1.04b	0.40b	0.18b
N_{140}	6.12ab	7.83a	7.53b	33.98c	15.70ab	9.24b	0.98b	0.38c	0.17c
N_{210}	6.44a	7.49a	7.56b	35.43c	15.03b	9.30b	1.05b	0.37c	0.17c
$S\bar{x}$	0.27	0.5	0.63	1.58	0.40	0.24	0.14	0.04	0.04
Nitrogen : Variety									
N_0:V_1	5.10c	5.67c	5.14e	42.47 a	15.18 ab	8.69 d	1.63 a	0.56 a	0.33 a
N_0:V_2	5.13c	5.80bc	5.06e	41.28 ab	15.24 ab	8.48 e	1.56 a	0.56 a	0.33 b
N_0:V_3	5.24bc	5.88bc	4.96f	40.13 bc	15.04 ab	8.23 f	1.52 a	0.55 a	0.31 c
N_{70}:V_1	5.81bc	7.97a	7.71a	38.34 cd	17.00 a	9.69 a	1.07 bc	0.41 b	0.18 d
N_{70}:V_2	5.85bc	7.97a	7.67ab	37.34 d	16.81 a	9.60 ab	1.03 c	0.40 b	0.17 d
N_{70}:V_3	5.91bc	7.94a	7.61bc	36.16 de	16.51 a	9.48 b	1.01 c	0.40 b	0.17 d
N_{140}:V_1	6.11b	7.84a	7.50d	34.85 ef	15.85 ab	9.26 c	1.02 c	0.38 bc	0.17 d
N_{140}:V_2	6.12b	7.84a	7.53cd	33.91 ef	15.71 ab	9.23 c	0.98 c	0.38 bc	0.17 d
N_{140}:V_3	6.13b	7.81a	7.56cd	33.19 f	15.55 ab	9.23 c	0.94 c	0.37 bc	0.16 e
N_{210}:V_1	7.11a	6.76b	7.56cd	38.18 cd	13.58 b	9.35 c	1.20 b	0.34 c	0.17 d
N_{210}:V_2	6.10b	7.85a	7.56cd	34.42 ef	15.82 ab	9.29 c	0.99 c	0.38 bc	0.17 d
N_{210}:V_3	6.10b	7.84a	7.56cd	33.68 f	15.71 ab	9.26 c	0.96 c	0.38 bc	0.17 d
$S\bar{x}$	0.16	0.27	0.33	0.91	0.26	0.13	0.07	0.02	0.02
ANOVA									
Variety (V)	NS	NS	*	**	NS	**	**	NS	**
N rate (N)	**	**	**	**	*	**	**	**	**
V X N	*	*	*	*	*	**	*	*	**
CV (%)	8.60	7.62	0.72	3.48	7.85	0.80	7.45	5.76	1.47

Means with the same letters or without letters within the same column do not differ significantly.

* = Significant at 5% level of probability, ** = Significant at 1% level of probability, NS = Not significant

V_1= BRRI dhan29, V_2= Hira-2, V_3= Binadhan-10

N_0= 0 Kg N ha-1, N_{70}= 70 Kg N ha^{-1}, N_{140}= 140 Kg N ha^{-1}, N_{210}= 210 Kg N ha^{-1}

Table S5. Yield and yield contributing parameters of three rice varieties under four N treatments attained from ANOVA

Variety	PH (cm)	TT (no.)	ET (no.)	PL (cm)	GP (no.)	TGW (g)	GY (g pot^{-1})	SY (g pot^{-1})	BY (g pot^{-1})	HI (%)
V_1	85.58 b	12.58 c	10.08 c	20.42 b	111.08 c	22.96 b	23.47 c	23.63 c	47.09 c	49.83
V_2	81.75 b	13.50 b	11.50 b	21.76 ab	113.67 b	23.65 ab	23.90 b	24.06 b	47.96 b	49.84
V_3	96.00 a	14.58 a	12.58 a	22.71 a	115.75 a	24.11 a	24.73 a	24.90 a	49.63 a	49.84
$S\bar{x}$	4.26	0.58	0.72	0.66	1.35	0.33	0.37	0.37	0.74	0.01
Nitrogen										
N_0	77.00b	8.11 d	6.44 c	18.66 c	104.22 d	20.98 d	18.91 c	19.03 c	37.94 c	49.84 a
N_{70}	90.89a	13.67 c	11.89 b	21.60 b	112.22 c	22.38 c	25.45 b	25.59 b	51.03 b	49.87 a
N_{140}	93.44a	17.22 a	14.33 a	23.86 a	120.00 a	26.23 a	26.40 a	26.58 a	52.98 a	49.84 a
N_{210}	89.78a	15.22 b	12.89 b	22.40 b	117.56 b	24.69 b	25.38 b	25.58 b	50.95 b	49.80b
$S\bar{x}$	3.67	1.96	1.72	1.09	3.49	1.17	1.72	1.74	3.46	0.01
Nitrogen : Variety										
N_0:V_1	70.33 h	6.67 d	5.33 g	17.84 h	102.33 h	21.24 fg	17.89 h	18.01 i	35.90 h	49.82 ab
N_0:V_2	72.00 h	8.67 c	6.00 g	19.50 fg	104.33 gh	21.44 e-g	18.63 g	18.75 h	37.38 g	49.83 a
N_0:V_3	88.67 f	9.00 cc	8.00 f	18.65 gh	106.00 g	20.26 g	20.22 f	20.33 g	40.55 f	49.86 a
N_{70}:V_1	89.00 ef	13.00 b	10.00 e	20.72 ef	109.00 f	21.88 e-g	25.17 e	25.29 f	50.46 e	49.87 a
N_{70}:V_2	84.67 g	13.33 b	12.67 cd	21.55 de	112.67 e	22.17 e-g	25.41 de	25.55 d-ff	50.97 de	49.86 a
N_{70}:V_3	99.00 b	14.67 b	13.00 b-d	22.54 b-d	115.00 de	23.10 d-f	25.77 cd	25.91 c-e	51.68 cd	49.86 a
N_{140}:V_1	92.33 cd	16.33 a	13.33 bc	22.24 c-e	117.33 cd	25.17 b-d	25.90 b-d	26.05 b-d	51.95 b-d	49.86 a
N_{140}:V_2	85.67 g	17.33 a	14.33 ab	23.51 bc	120.33 ab	26.01 ab	26.35 b	26.52 b	52.86 b	49.84 a
N_{140}:V_3	102.33 a	18.00 a	15.33 a	25.82 a	122.33 a	27.52 a	26.96 a	27.16 a	54.13 a	49.82 ab
N_{210}:V_1	90.67 de	14.33 b	11.67 d	20.88 ef	115.67 d	23.54 c-e	24.92 e	25.15 f	50.06 e	49.77 b
N_{210}:V_2	84.67 g	14.67 b	13.00 b-d	22.49 bc	117.33 cd	24.98 b-d	25.22 e	25.41 ef	50.63 e	49.82 ab
N_{210}:V_3	94.00 c	16.67 a	14.00 a-c	23.83 b	119.67 bc	25.56 a-c	25.99 bc	26.18 bc	52.17 bc	49.81 ab
$S\bar{x}$	2.73	1.06	0.96	0.66	1.92	0.65	0.92	0.92	1.84	0.01
ANOVA										
Variety (V)	**	**	**	**	**	*	**	**	**	NS
N rate (N)	**	**	**	**	**	**	**	**	**	**
V x N	**	*	*	*	*	*	**	**	**	*
CV (%)	1.26	7.06	6.71	3.88	1.24	5.05	1.18	1.23	1.20	0.06

Means with the same letters or without letters within the same column do not differ significantly.

** = Significant at 1% level of probability, * = Significant at 5% level of probability, NS = Not significant

V_1= BRRI dhan29, V_2= Hira-2, V_3= Binadhan-10

N_0= 0 Kg N ha^{-1}, N_{70}= 70 Kg N ha^{-1}, N_{140}= 140 Kg N ha^{-1}, N_{210}= 210 Kg N ha^{-1}
