# Supplementary figures and images for: Optimum Nitrogen Application Acclimatizes Root Morpho-Physiological Traits and Yield Potential in Rice under Subtropical Conditions
Source: Life (Basel). 2022 Dec 7;12(12):2051. doi: 10.3390/life12122051 (PMC9786123; doi:10.3390/life12122051)

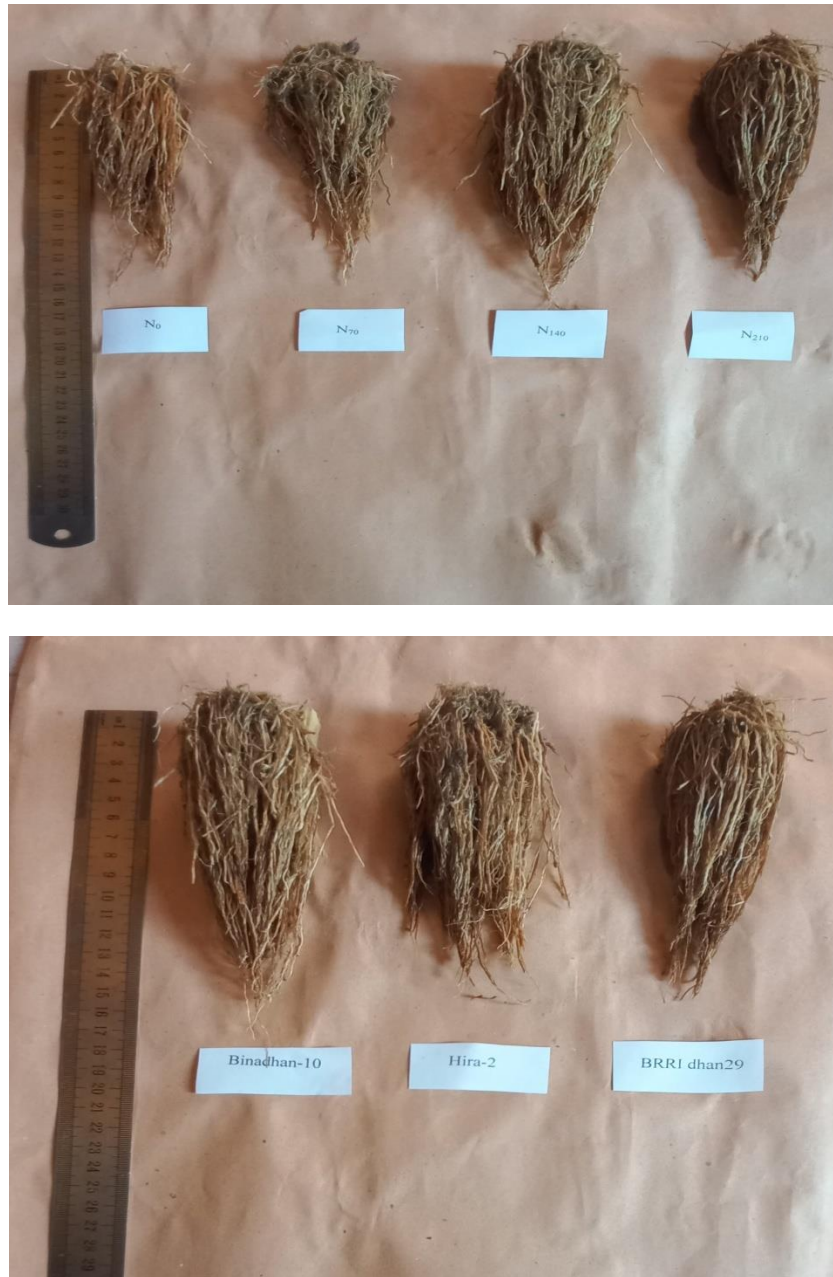

**Figure S1.** Root length measurements showing variation among varieties and N conditions

Supplement: Supplementary file 1 [file life-12-02051-s001.zip › Supplementary Figure S1.pdf]

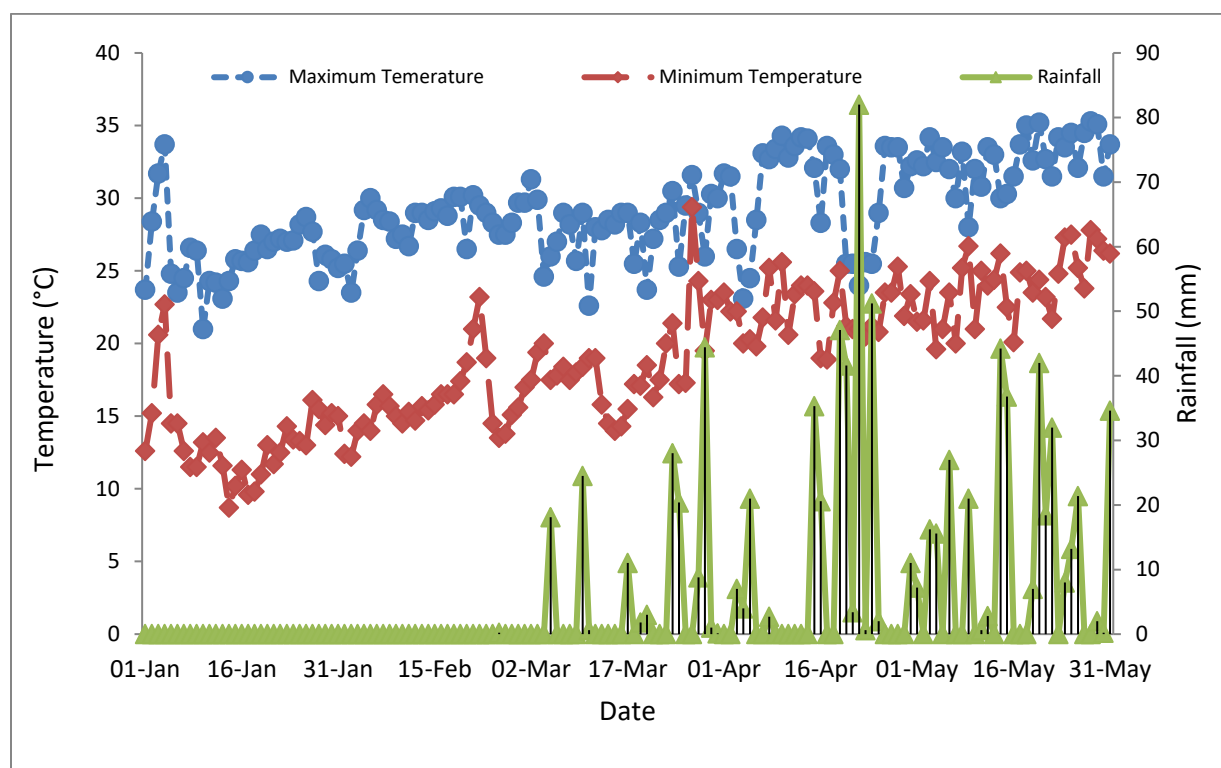

**Figure S2.** Weather condition during crop growth stages of *boro* rice

Supplement: Supplementary file 1 [file life-12-02051-s001.zip › Supplementary Figure S2.pdf]
